# A Comparative Study of Conventional Versus Ultrasound-Assisted Femoral Arterial Cannulation: A Prospective Randomized Trial

**DOI:** 10.7759/cureus.80871

**Published:** 2025-03-20

**Authors:** Mayank K Singhal, Virendra Kumar, Shilpi Misra, Praveen K Das, Manoj Giri, Suraj Chaudhary

**Affiliations:** 1 Anesthesiology, Shaikh-Ul-Hind Maulana Mahmood Hasan (SMMH) Medical College, Saharanpur, IND; 2 Anesthesiology and Critical Care, Dr Ram Manohar Lohia Institute of Medical Sciences (RMLIMS), Lucknow, IND

**Keywords:** anatomy, bleeding, catheterization, femoral artery, ultrasound, vascular complications

## Abstract

Background*:* The common femoral artery (CFA) is one of the most frequently used vascular access sites for diagnostic and therapeutic coronary interventions. This study aimed to compare the traditional and ultrasound (US)-assisted methods for CFA cannulation, focusing on the incidence of complications.

Methods:* *This study included 100 participants, who were divided into two groups (Group C and Group U), each consisting 50 participants. Group C underwent CFA catheterization using the conventional method, while Group U underwent US-guided CFA catheterization. The following parameters were recorded for both groups: time taken for cannulation, first-pass success rate, number of skin punctures required, and complications such as posterior wall puncture, hematoma formation, femoral vein puncture, pseudoaneurysm formation, arteriovenous fistula, and retroperitoneal hemorrhage.

Results: The mean time for cannulation in the conventional group was 100.90 seconds, compared to 78.16 seconds in the US-guided group. The mean number of skin punctures was 1.36 in Group U and 1.64 in Group C. The mean first-pass success rate was 64% in Group U and 42% in Group C. No incidents of posterior wall puncture were observed in Group U, while there was one incident in Group C. The incidence of femoral vein punctures was lower in Group U than in Group C.

Conclusion: The study findings suggest that US-guided CFA cannulation is quicker, requires fewer attempts, and results in fewer skin punctures compared to the conventional method.

## Introduction

Intra-arterial cannulation is an invasive method for measuring blood pressure and mean arterial pressure more accurately than noninvasive methods [1]. Various sites for arterial cannulation can be used, including the radial, posterior tibial, femoral, brachial, axillary, ulnar, dorsalis pedis, and temporal arteries [2]. The common femoral artery (CFA) is one of the most frequently used vascular access sites for diagnostic and therapeutic coronary interventions [3]. The growing popularity of CFA cannulation is due to its lower risk of thrombosis and accidental catheter dislodgment [4]. Despite its widespread use, CFA cannulation is not without complications. Although less frequent, complications can include local hematoma, retroperitoneal hemorrhage, pseudoaneurysm, arteriovenous fistula, infection, and injury to surrounding structures [5]. These complications may even present years after the procedure [6]. The ideal site for femoral arterial puncture (excluding skin puncture) is located approximately 1 cm lateral to the most medial aspect of the femoral head, midway between its superior and inferior borders (Rupp’s rule) [7]. Cannulation below the femoral artery bifurcation, where the femoral sheath lacks scaffolding, is associated with higher complication rates [8]. Pseudoaneurysm and arteriovenous fistula formations are more common when the puncture site is below the inguinal ligament. In contrast, the risk of retroperitoneal hemorrhage increases when the puncture site is above the inguinal ligament [9]. Several randomized clinical trials have evaluated the efficacy and safety of ultrasound (US) guidance in patients undergoing percutaneous cardiovascular interventions (PCVIs) in recent years, providing new insights into the role of new technologies in invasive procedures [10]. Real-time US guidance facilitates femoral arterial access and reduces vascular complications [11]. It is particularly helpful in cases of shock, hypotension, obesity, coagulopathy, or when palpation fails to locate the artery [12]. However, these trials have often not focused on major vascular complications as primary outcomes, and the impact of US guidance on clinically significant vascular access-related complications during femoral artery catheterization remains unclear. These gaps in the literature highlight the need for further studies assessing the efficacy and safety of US-guided cannulation in different interventional vascular procedures, with a specific focus on vascular access-related complications during CFA catheterization.

Hence, the present study aims to compare the efficacy and safety of conventional versus US-assisted CFA cannulation.

## Materials and methods

Study design

A prospective, randomized, comparative interventional study was conducted at Dr Ram Manohar Lohia Institute of Medical Sciences, Vibhuti Khand, Gomti Nagar, Lucknow, Uttar Pradesh, India, from March 2020 to August 2021 (520 days). The study aimed to compare the effectiveness and safety of US-guided versus conventional landmark-based techniques for CFA cannulation.

Study population

The study included individuals aged 18-60 years, of either sex, with normal coagulation profiles, scheduled for routine cardiac surgery, or undergoing routine angiography requiring CFA cannulation. Exclusion criteria included subjects with abnormal coagulation profiles, pregnant women, individuals who had undergone CFA catheterization within the last seven days, and patients undergoing emergency cardiac surgery. Additionally, patients undergoing coronary artery bypass grafting (CABG) were not included.

Ethical consideration

The clinical research was conducted in accordance with the ethical principles for medical research involving human subjects, as outlined in the Helsinki Declaration of 2013. After obtaining institutional ethical clearance from the Institutional Ethical Committee (IEC no. 62/18) and written consent from the study participants, 100 subjects were randomly assigned to Groups C and U, with 50 participants in each group. This study was registered with the Clinical Trials Registry-India (CTRI/2021/02/031328).

Randomization and allocation

Patients were randomly assigned to either the conventional landmark-guided (Group C) or the US-guided (Group U) group using computer-generated random numbers. Block randomization (block size 4) was employed to ensure equal distribution. Allocation concealment was maintained through the use of sealed opaque envelopes, which were opened just before the procedure.

Procedure details

Patients were randomly allocated to Group C (participants undergoing CFA catheterization using the conventional method) and Group U (subjects undergoing CFA catheterization under US guidance).

All eligible patients were thoroughly assessed in the pre-anesthetic check-up clinic (PAC), and those cleared in PAC were scheduled for femoral arterial cannulation. Arterial catheterizations were performed using standard sterile precautions using the Seldinger technique in both groups. All equipment, including monitoring devices and the US machine (Sonosite M-Turbo, FUJIFILM Sonosite, Bothell, WA, US), was prepared, and the transducer was zeroed before use. Chlorhexidine-alcohol skin antiseptic solution was applied to the access site and allowed to dry. Sterile gloves were worn, and once the antiseptic solution dried, a fenestrated drape was applied and positioned. For all CFA catheterization procedures, the patient was positioned supine, with the right femoral artery selected for cannulation. The leg was comfortably abducted and externally rotated. On alternate days, one operator worked in the cardiovascular and thoracic surgery operating theater (CVTS OT) and the cardiac catheterization laboratory. Two right-handed operators with at least 100 prior CFA cannulation procedures performed the procedures.

Conventional Landmark-Guided Technique (Group C)

The operators used their nondominant (left) hand to gently palpate the artery, while the dominant (right) hand was used for catheterization. The needle was held in the right hand, and the artery was palpated with the left hand, 2.5 cm below the midpoint of the inguinal ligament. The CFA was always punctured in the femoral triangle, 2.5 cm below the inguinal ligament. The needle was inserted at a 45-degree angle to the skin at the palpated location. A Leadercath 2 E.L. (Vygon, Swindon, UK) 16-gauge arterial line was used, consisting of a 100-mm needle, a 15-cm catheter, and a guidewire with an outer diameter of 0.97 mm and a length of 46 cm. The procedure was performed with the Seldinger technique. While stabilizing the intravascular catheter with the nondominant hand, the dominant hand was used to remove the needle from the intravascular catheter. 

Ultrasound-Guided Technique (Group U)

The operators used the US to identify the patent CFA and guide catheter placement. A linear high-frequency probe (5-10 Hz) with a 38 mm footprint was employed. Initially, the short-axis (cross-sectional) view was used to aim the needle at the artery. The probe was then rotated 90 degrees to achieve the long-axis (in-plane longitudinal) view of the needle and the artery, which provides better imaging of arterial puncture and catheter placement and helps avoid posterior wall puncture. A sterile cover was placed over the probe, which was held in the nondominant (left) hand, and positioned 2.5 cm inferior to the midpoint of the inguinal ligament. The probe was moved transversely as needed to center the artery on the US screen, after which cannulation of the femoral artery was performed under US guidance in the long-axis view. The vessels were visualized, with the guidewire extended 2 cm beyond the vessel. The needle was traced and punctured carefully as it approached the vessel to avoid injury to the posterior wall. After puncture, the guidewire was inserted, and a US screenshot was taken to document the vessel and the guidewire for later measurement. Accurate distances were measured in the long-axis view. The correct arterial placement was confirmed by free arterial backflow and waveform analysis using an arterial pressure monitor.

Study outcomes

The primary outcome of this study was the occurrence of complications, including posterior wall puncture, hematoma formation, femoral vein puncture, pseudoaneurysm formation, and retroperitoneal hemorrhage. Secondary outcomes included time taken for cannulation (starting from needle puncture), first-pass success rate, and the number of skin punctures required. Hematoma formation around the puncture site was assessed immediately after successful cannulation, six hours after cannulation, and 24 hours after cannulation. For complications such as pseudoaneurysm, participants were monitored for seven days or contacted by phone if discharged earlier. In this study, the operators did not follow the de-cannulation process, and observation was limited to 24 hours following CFA cannulation, focusing solely on the cannulation procedure.

Sample size

Fifty participants were included in each group to test the null hypothesis that the proportion of patients with complications is equal between the groups, with a power of 0.8. The Type 1 error probability for this hypothesis was set at 0.05. There were no dropouts in the study.

Statistical analysis

All statistical analyses were performed using IBM SPSS Statistics for Windows, Version 26.0 (Released 2019; IBM Corp., Armonk, NY, US). Comparisons between groups at different time intervals were made using the Student t-test, while the chi-square test was used to compare all categorical and parametric data. A p-value of <0.05 was considered statistically significant. Time intervals were measured in seconds, with means reported to two decimal places.

## Results

Results were compared between the control group (Group C) and the study group (Group U), each with 50 participants. Group C consisted of 66% female and 34% male participants, while Group U consisted of 58% female and 42% male participants. There was no significant difference in sex distribution between the groups, indicating comparability. Table 1 presents the distribution of anthropometric parameters: height and weight. The mean height in Group C was 155.42 cm, while in Group U, it was 152.48 cm; this difference was statistically insignificant (p = 0.090). The mean weight in Group C was 52.90 kg, and in Group U, it was 52.45 kg; this difference was also statistically insignificant (p = 0.785). Table 2 displays coagulation parameters, with the mean prothrombin time (PT) of 12.15, the mean international normalized ratio (INR) of 1.27, and the mean platelet count of 184.5. No significant differences were observed in platelet count (p = 138), PT (p = 0.905), and INR (p = 0.368). Table 3 compares cannulation parameters. The mean cannulation time in the conventional group was 100.90 seconds, compared to 78.16 seconds in the US-guided group, showing a significant difference (p = 0.001). The mean number of skin punctures required for cannulation was 1.36 in the US-guided group and 1.64 in the conventional group, also showing a significant difference (p = 0.018). Table 4 shows the mean first-pass success rate: 32 for US-guided cannulation compared to 21 for the conventional group, demonstrating a significant difference (p = 0.028). Table 5 presents the mean angle of insertion, which was 45.26 degrees in the conventional group and 37.48 degrees in the US-assisted group. This difference was highly significant between (p < 0.001). 

**Table 1:** Between-group comparisons of anthropometric parameters Values are expressed as mean and standard deviation (SD). p < 0.05 was considered statistically significant. t: Student t-test.

S. N.	Parameter	Group C (n = 50)				Group U (n = 50)				t	Significance (p-value)
		Min.	Max.	Mean	SD	Min.	Max.	Mean	SD		
1	Height (cm)	142.0	170.0	155.42	7.81	123.0	170.0	152.48	9.30	1.712	0.090
2	Weight (kg)	40.00	72.00	52.90	7.17	38.00	72.00	52.49	7.72	0.274	0.785

**Table 2:** Between-group comparisons of laboratory parameters Values are expressed as mean and standard deviation (SD). p < 0.05 was considered statistically significant. PT: prothrombin time, INR: international normalized ratio, t: Student t-test.

S. N.	Parameter	Group C (n = 50)				Group U (n = 50)				t	Significance (p-value)
		Min.	Max.	Mean	SD	Min.	Max.	Mean	SD		
1	Platelet count (10^3^/uL)	104.0	352.0	184.50	56.60	95	267	169.68	41.24	1.496	0.138
2	PT (seconds)	10.0	14.9	12.15	1.38	10.0	19.3	12.19	1.88	-0.120	0.905
3	INR	0.87	1.57	1.22	0.19	0.86	1.60	1.18	0.19	0.904	0.368

**Table 3:** Between-group comparisons of cannulation parameters Values are expressed as mean and standard deviation (SD). p < 0.05 was considered statistically significant. t: Student t-test.

S. N.	Parameter	Group C (n = 50)		Group U (n = 50)		t	p-value
		Mean	SD	Mean	SD		
1	Time for cannulation (seconds)	100.90	17.82	78.16	14.56	6.988	<0.001
2	Number of skin puncture	1.64	0.66	1.36	0.48	2.411	0.018

**Table 4:** Between-group comparisons of first-pass success rate Values are expressed as mean and standard deviation (SD). p < 0.05 was considered statistically significant. χ^2^: chi-square test.

S. N.	Parameter	Group C (n = 50)		Group U (n = 50)		χ^2^	p-value
		No.	%	No.	%		
1	First-pass success	21	42.0	32	64.0	4.857	0.028

**Table 5:** Between-group comparisons of the angle of insulation Values are expressed as mean and standard deviation (SD). p < 0.05 was considered statistically significant. t: Student t-test.

S. N.	Parameter	Group C (n = 50)				Group U (n = 50)				t	Significance (p-value)
		Min.	Max.	Mean	SD	Min.	Max.	Mean	SD		
1	Angle of insinuation (degrees)	42	48	45.26	1.736	32	44	37.48	3.02	-15.79	<0.001

As shown in Table 6, there was no incidence of posterior wall puncture in the US-guided group, compared to one incident in the conventional group. However, this difference was not statistically significant (p > 0.05). The incidence of femoral vein punctures was 4% in the US-guided group and 28% in the conventional group, which was statistically significant (p = 0.022). Fortunately, other complications, such as pseudoaneurysms and retroperitoneal hemorrhage, did not occur in either group.

**Table 6:** Between-group comparisons of other complications Values are expressed as numbers and percentages. p < 0.05 was considered statistically significant. χ^2^: chi-square test.

S. N.		Group C (n = 50)		Group U (n = 50)		Statistics	
		Number	%	Number	%	χ^2^	Significance (p-value)
1	Post-wall puncture	1	2.0	0	0.0	1.010	0.315
2	Femoral vein puncture	14	28.0	2	4	10.71	0.001

Table 7 compares hematoma formation at different time intervals. The incidence of immediate postoperative hematoma formations was 12% in the US-guided group compared to 28% in the conventional group, with a statistically significant difference (p = 0.046).

**Table 7:** Between-group comparisons of hematoma formation at different time intervals Values are expressed as numbers and percentages. p < 0.05 was considered statistically significant. χ^2^: chi-square test.

S. N.	Time interval	Group C (n = 50)		Group U (n = 50)		Statistics	
		Number	%	Number	%	χ^2^	Significance (p-value)
1	Immediate postoperative period	14	28.0	6	12.0	4.000	0.046
2	After six hours	11	22.0	6	12.0	1.772	0.183
3	After 24 hours	8	16.0	5	10.0	0.796	0.372
4	After 48 hours	3	6.0	0	0.0	3.093	0.079

## Discussion

Statistical analysis revealed no significant difference in anthropometric (height and weight) and laboratory parameters (PT, INR, and platelet counts) between Group C and Group U, as shown in Table 1 and Table 2, respectively. Therefore, the groups were comparable based on these measurements. A significant difference was observed in the time required for cannulation, with the conventional group taking a mean of 100.90 seconds compared to 78.16 seconds in the US-guided group (p < 0.001), as shown in Table 3. Additionally, the mean number of skin punctures required for cannulation was 1.36 in the US-guided group compared to 1.64 in the conventional group, demonstrating a significant difference (p < 0.018), as shown in Table 3.

The mean first-pass success rate for US-guided cannulations was 32, compared to 21 in the conventional group, demonstrating a significantly higher success rate in the US-guided group (p < 0.028), as shown in Table 4. Gedikoglu et al. [13] compared US guidance with the traditional palpation method for CFA puncture. A total of 208 patients were randomized into two groups: one underwent a US-guided puncture, and the other had a palpation-guided puncture. The time for successful US-guided cannulation was significantly shorter (53 seconds) than the palpation-guided method (74 seconds) (p < 0.003). The current study also found a shorter time for cannulation in Group U [14]. Kurisu et al. [15] conducted a prospective observational cohort study at Hokkaido University Hospital in 2014, enrolling 64 patients requiring CFA access for diagnostic and interventional neurovascular procedures. They compared the occurrence of hematoma in the conventional group versus the US-guided group. Their results showed that US guidance was beneficial in visualizing CFA, which was difficult to detect by usual palpation. Under US-guided CFA access, the median number of attempts was one, and the first-pass success rate was 63.6%. These findings align with the outcomes of the current study, as shown in Table 3 and Table 4, where Group U had fewer attempts and a significantly higher first-pass success rate (p < 0.05). In both methods, the angle of insertion was measured using ultrasonic images after placing the guidewire. Table 5 shows a significant difference in the angle of insertion between Group C and Group U, with the US-assisted group having a smaller angle of insertion (p = 0.001).

Sorrentino et al. [16] conducted a meta-analysis of randomized controlled trials following PRISMA guidelines. They searched Medline, Embase, Scopus, and oral presentations from recent international conferences for studies published or posted until May 30, 2019. Seven trials were included, with 3,180 patients randomized to either the US group (n = 1,564) or the standard approach group (n = 1,616). The first-attempt success rate was significantly higher in the US group (82.0%) than the standard approach group (58.7%) (RR 1.36, 95% CI 1.17-1.57, p < 0.0001). Time to access was also significantly shorter in the US group (95% CI −0.28 to −0.10, p < 0.0001), along with fewer attempts required (p < 0.0001). The current study supports these findings. They also reported a lower risk of access-site hematoma in the US group (1.2% vs. 3.3%, CI 95% 0.20-0.83, p = 0.01), which aligns with the results observed in the present study, as shown in Table 6 and Table 7. In this study, complications such as pseudoaneurysm and retroperitoneal hemorrhage did not occur in either group. Although hematoma formation was less frequent in the US group at 6, 24, and 48 hours, the difference was not statistically significant.

Marquis-Gravel et al. [17] conducted a randomized controlled trial to compare US-guided versus anatomical landmark approaches for CFA access in coronary angiography. A total of 129 patients were randomized (64 to the US-guided group and 65 to the anatomical landmark group). Additionally, four other studies met the inclusion criteria and were included in the meta-analysis, involving 1,553 patients. After pooling the data, the rate of venipuncture was significantly lower in the US-guided group (p < 0.0001). The present study shows results similar to those of the US-guided group, as shown in Table 5. The incidence of immediate postoperative hematoma formations was 12% in the US group, compared to 28% in the conventional group, with a significant difference (p = 0.046), as shown in Table 7.

Limitations of study

While the present study is valuable in assessing the benefits of US-guided CFA cannulation, it has several limitations. These include small sample size, lack of diversity in the patient population, potential patient selection bias, challenges with blinding, randomization issues in small groups, subjective measures, and its single-center design. Therefore, a multicenter study is needed to address these limitations.

## Conclusions

In conclusion, US-guided cannulation of the CFA was found to be more accessible, requiring fewer attempts, less time, fewer skin punctures, and resulting in fewer complications, such as hematoma formation, posterior wall puncture, and femoral vein puncture, compared to the conventional method. Additionally, it was associated with a smaller angle of insertion. Further studies using randomized allocation in age-matched patients are recommended to draw more robust conclusions.
